# Psychosocial factors affecting resilience in Nepalese individuals with earthquake-related spinal cord injury: a cross-sectional study

**DOI:** 10.1186/s12888-018-1640-z

**Published:** 2018-03-02

**Authors:** Muna Bhattarai, Khomapak Maneewat, Wipa Sae-Sia

**Affiliations:** 1 Hope International College, Kathmandu, 44600 Nepal; 2 Faculty of Nursing, Prince of Songkla University, Hat Yai, Songkhla Thailand

**Keywords:** Resilience, Social support, Self-efficacy, Spirituality, Depressive mood, Earthquake, Spinal cord injury, Nepal

## Abstract

**Background:**

One of many types of injuries following an earthquake is spinal cord injury (SCI) which is a life-long medically complex injury and high-cost health problem. Despite several negative consequences, some persons with SCI are resilient enough to achieve positive adjustment, greater acceptance, and better quality of life. Since resilience is influenced by several factors and can vary by context, it is beneficial to explore factors that affect the resilience of people who sustained spinal cord injury from the 2015 earthquake in Nepal.

**Methods:**

A descriptive cross-sectional study included 82 participants from the Spinal Injury Rehabilitation Center and communities in Nepal. Participants completed the Demographic and Injury-related Questionnaire, Connor-Davidson Resilience Scale, Multidimensional Scale of Perceived Social Support, Moorong Self-efficacy Scale, Intrinsic Spirituality Scale, and Patient Health Questionnaire-9. Pearson’s correlation and point biserial correlation analyses were performed to examine associations between resilience and independent variables. A hierarchical regression analysis was used to identify the influence of certain factors.

**Results:**

Findings indicated significant associations between resilience and social support (*r* = 0.42, *p* < 0.001), self-efficacy (*r* = 0.53, *p* < 0.001), depressive mood (*r* = − 0.50, *p* < 0.001) and demographic variables which included sex (*r* = 0.47, *p* < 0.001), employment (*r* = 0.27, *p* = 0.016), and current living location (*r* = 0.24, *p* = 0.029). There was a non-significant association between resilience and spirituality (*r* = − 0.12, *p* > 0.05). In hierarchical regression analysis, an overall regression model explained 46% of the variance in resilience. Self-efficacy (β *=* 0.28, *p* = 0.007) and depressive mood (β *=* − 0.24, *p* = 0.016) significantly determined resilience after controlling the effect of demographic variables. Among the demographic factors, being male significantly explained the variance in resilience (β *=* 0.31, *p* = 0.001).

**Conclusions:**

Multiple psychosocial and demographic factors were associated with resilience in people who sustained an earthquake-related SCI. Mental health professionals should demonstrate concern and consider such factors in allocating care in this group. Development of intervention research concerning resilience is recommended to strengthen resilience in order to improve rehabilitation outcomes and enhance reintegration of individuals with SCI into their communities.

## Background

Earthquakes are devastating natural disasters that cause catastrophic impacts and extensive damage to property, residences, economics, physical and psychological health, and even human life [1]. Among the neurological injuries following an earthquake, spinal cord injury (SCI) is a life-long medically complex injury and high-cost health problem that affects physical, psychological and social health, and the well-being of an individual [2]. In Nepal, an earthquake of 7.8 magnitude and a subsequent aftershock of 7.3 magnitude in April and May 2015 left more than 173 Nepalese people suffering from SCI [3].

SCI causes complete or incomplete loss of motor and sensory functions including muscle paralysis, impairment of bladder and bowel control, and sexual function [4]. Along with physical complications, the psychosocial consequences that follow SCI are anxiety, depression, social withdrawal, low self-confidence, post-traumatic stress disorder, and suicidal thoughts and attempts. As a result, these conditions further affect and worsen rehabilitation and overall health [5–7]. Individuals who sustained disabilities, including SCI from an earthquake, reported higher psychological distress than the normal survivors of an earthquake [8]. Thus, both the earthquake and SCI are stressful traumatic events that have numerous negative consequences on survivors.

Resilience or the ability of an individual to thrive in the face of adversity [9] plays a vital role among people with SCI to overcome the catastrophic changes and negative impacts from the consequences of SCI [10, 11]. Resilience can be defined as the ability of a person with SCI to bounce back from a stressful experience and adapt to the changes resulting from a SCI [12]. Research showed that 66% of individuals who sustained a SCI were resilient [13]. As a result, they achieved positive adjustment, greater acceptance, and a better quality of life [10, 13]. However, about 34% had significant problems with resilience such as depressed mood or low self-efficacy [13]. It was recognized that resilience is influenced by numerous factors. Factors contributing to resilience can vary and have different impacts on individuals in different cultures, societies, and geographical regions or contexts [14, 15].

Several factors have been explored and evidenced to either enhance or impede resilience in persons with SCI and victims of earthquakes [6, 16–18]. Previous studies revealed that self-efficacy, social support [13, 19], and spirituality [11, 20, 21] enhanced resilience in persons with SCI, whereas depression/depressive mood [6, 11, 16, 19], stress, anxiety, and internal locus of control [6] impeded resilience of those individuals. Previous findings regarding the influence of demographic and injury-related factors on resilience are still inconclusive [6, 13, 16, 22]. Most of the aforementioned factors were also found to affect resilience in individuals surviving earthquakes or other disasters [17, 18, 23]. Nonetheless, there is still a dearth of studies exploring factors affecting resilience of persons who sustained SCI from disasters including earthquakes.

In addition, previous studies were conducted regarding resilience factors among people with SCI and survivors of earthquake but mostly in developed countries [6, 13, 17]. This leads to the question of generalizability of those results in the context of Nepal. Nepal is a developing country which has a different culture, geographical distribution, and healthcare system. Nepal is a collectivist society and many Nepalese live in rural areas that lack the availability of healthcare services [24]. Thus, factors affecting resilience among Nepalese with earthquake-related SCI could be different from people in other geographical and cultural contexts. This study hypothesized that psychosocial factors, i.e., social support, self-efficacy, spirituality, and depressive mood, determine resilience among Nepalese with the 2015 earthquake-related SCI.

## Methods

### Design and setting

This descriptive cross-sectional study was carried out in Nepal between December 2016 and February 2017. The participants were recruited from the Spinal Injury Rehabilitation Center (SIRC) in Kavre district, which is the major rehabilitation center for people with SCI in Nepal, and in community settings. The communities included eight districts of Nepal which were affected by the 2015 earthquake: Kathmandu, Lalitpur, Bhaktapur, Kavre, Sindupalchowk, Gorkha, Dhading, and Nuwakot.

### Participants

Inclusion criteria for the participants were: (1) individuals who sustained SCI from the 2015 Nepal earthquake; (2) aged 18 years or older; (3) had been admitted to the SIRC for rehabilitation; (4) living in the community of eight districts after discharge from the SIRC; (4) understand and speak the Nepali language; and (5) conscious and absence of severe cognitive impairment (based on information obtained from the medical records and history taking). Of 117 patients with earthquake-related SCI admitted to the SIRC, the list and contact details of 101 individuals were obtained. The sample size of equal to or greater than 50 + (8 times the number of factors) was calculated based on the sample size calculation technique of multiple regression [25]. Therefore, 82 participants were recruited in this study using convenience sampling. Six participants who were readmitted for management of complications were included from the SIRC. Another 76 eligible participants were contacted via cell phone. Since all of them agreed to participate, they were approached in their communities. Written informed consent was obtained from each participant. Data collection was done via interview or self-administration of questionnaires depending on the ability of the participants to read and write.

### Measurements

The demographic and injury-related questionnaire was developed by the researchers. The demographic variables included sex, ethnicity, current living location, marital status, education, employment, income, and types of caregiver. The injury-related variables comprised of injury level, completeness of injury, chronic disease, and complications related to SCI. Data related to injury level, completeness of injury, chronic disease, and complications were retrieved from the medical records, interview, observation, and physical examination.

Five standardized research instruments were used for data collection. These tools were translated into the Nepali language using the back translation process [26]. The Connor-Davidson Resilience Scale (CD-RISC) was used to measure resilience. The CD-RISC examines resilience as the ability to cope in the face of adversity and is comprised of five factors: (1) personal competence, high standards, and tenacity; (2) trust in one’s instincts, tolerance of negative affect, and strengthening effects of stress; (3) positive acceptance of change and secure relationships; (4) control; and (5) spiritual influences. The CD-RISC consists of 25 items scored on a 5-point Likert scale which ranges from 0 (not true at all) to 4 (true nearly at all of the time). The range of total possible scores is from 0 to 100, where higher scores indicate greater resilience [9]. The tool has demonstrated adequate validity and reliability among persons with SCI [11] and victims of an earthquake [18]. The present study reported a Cronbach’s alpha of 0.82 for the Nepali version of the CD-RISC.

The Multidimensional Scale of Perceived Social Support (MSPSS) was used to measure social support. The MSPSS measures perceived social support from three sources: family, friends, and significant other [27]. The tool consists of 12 items scored on a 7-point Likert scale ranging from 1 (very strongly disagree) to 7 (very strongly agree). Total scores range from 12 to 84, where higher scores represent greater perceived social support. Evidence of validity and reliability has been provided with adequate Cronbach’s alpha among people with SCI [28]. The present study demonstrated a Cronbach’s alpha of 0.89.

The Moorong Self-efficacy Scale (MSES) was used to measure self-efficacy in performing functional, social, leisure and vocational activities post-SCI [29, 30]. The MSES is comprised of 16 items with a 7-point semantic differential scale ranging from 1 (very uncertain) to 7 (very certain). The total scores range from 16 to 112, where higher scores indicate greater perceived self-efficacy. The MSES has demonstrated good validity and reliability in outpatient and inpatient individuals with SCI in a previous study [29] with a Cronbach’s alpha of 0.79 in the present study.

The Intrinsic Spirituality Scale (ISS) was used to measure the spirituality. The ISS is comprised of six items and each item consists of phrase completion. The participants are asked to complete a phrase by selecting an option from a list of 11 responses ranging from 0 to 10. The score is calculated by summing the scores of the six items and dividing the total score by six. The ISS is considered an appropriate measure to assess intrinsic spirituality [11]. Cronbach’s alpha was 0.76 in this study.

Depressive mood was assessed using the Patient Health Questionnaire-9 (PHQ-9) [31]. This tool consists of nine items based on diagnostic criteria of Diagnostic and Statistical Manual of Mental Health Disorder-IV for depressive disorder. The response is rated in a 4-point Likert scale ranging from 0 (not at all) to 3 (nearly every day). The total score can range from 0 to 27, where higher scores represent a higher depressive mood. The PHQ-9 has been standardized with sound psychometric properties [16, 22] and Cronbach’s alpha was 0.87 in this study.

### Statistical analyses

The analyses were performed using the Statistical Package for the Social Sciences Software (SPSS; version 16.0). All study variables met the assumptions of correlation and multiple regression analysis. Missing data were estimated by expectation maximization at the individual item level since data were found to be missing completely in a random pattern (Little’s MCAR chi-square = 15.70, *p* = 0.20). Descriptive statistics were used to examine demographic and injury-related data. Correlations between resilience and independent variables were tested by a point-biserial correlation or Pearson’s correlation depending on the level of measurement of each variable. A hierarchical regression analysis was performed to identify a unique contribution of each variable in determining resilience. Demographic and injury-related variables that were significantly correlated with resilience were entered into the first block to control for the confounding effects. Then, self-efficacy, depressive mood, and social support were entered into the second, third, and fourth blocks, subsequently based on the theoretical predictions. A statistical level of significance was set at a *p* value of < 0.05.

## Results

Participants included 34 females with a mean age of 37.15 years (SD 12.63) and 48 males with a mean age of 33.15 (SD 10.22). Sixteen participants (19.5%) had chronic disease which included hypertension (*n* = 7), asthma, gout, and gastrointestinal problems. The reported SCI-related complications were pain (*n* = 66), spasticity (*n* = 21), pressure injury (*n* = 10), and urinary tract infection (*n* = 8) [32]. Table 1 shows the demographics and injury-related characteristics of the participants.

**Table 1:** Demographic and injury-related characteristics of participants (*N* = 82)

Variables		Number	Percent
Age (years) M = 34.80, SD = 11.38, Min-Max = 18–64			
Sex	Female	34	41.5
	Male	48	58.5
Ethnicity	Aryan	30	36.6
	Mongol	52	63.4
Current living location	Rural	51	62.2
	Urban	31	37.8
Marital status	Unmarried	32	39.0
	Married/Divorced	50	61.0
Education	Primary or lower	42	51.2
	Secondary or higher	40	48.8
Employment	Unemployed	61	74.4
	Employed	21	25.6
Income^a^	No or > Rs. 10,000	67	81.7
	≤ Rs. 10,000	15	18.3
Injury level	Tetraplegia	4	4.9
	Paraplegia	78	95.1
Completeness of injury	Complete	29	35.4
	Incomplete	53	64.6
Chronic disease	No	66	80.5
	Yes	16	19.5
Complication related to SCI	No	9	11.0
	Yes	73	89.0
Caregiver	Family members	69	84.1
	Organizational caregiver	13	15.9

Note. ^a^1USD = Nepali Rupee (Rs.) 106.78

The average resilience score of the participants was 64.76 (SD 14.02). Descriptive statistics for social support, self-efficacy, spirituality, and depressive mood are presented in Table 2. A point-biserial correlation analysis revealed a significant correlation between sex and resilience (*r* = 0.47, *p* < 0.001). Likewise, a significant positive association was found between employment and resilience (*r* = 0.27, *p* = 0.016). In addition, current living location of the participants was positively associated with resilience (*r* = 0.24, *p* = 0.029). There were no significant associations between resilience and other demographic and injury-related variables (Table 3).

**Table 2:** Resilience, social support, self-efficacy, spirituality, and depressive mood of participants

Measures	Mean	SD	Possible score	Actual score
Resilience (CD-RISC)	64.76	14.02	25–100	33–95
Social support (MSPSS)	62.74	10.74	12–84	36–84
Self-efficacy (MSES)	81.63	16.88	16–112	41–111
Spirituality (ISS)	6.17	2.91	0–10	0–10
Depressive mood (PHQ-9)	8.76	5.30	0–27	0–25

**Table 3:** Correlations between resilience and demographic and injury-related variables

Demographic and injury-related variables	Correlation with resilience	
	*r*	*P*
Age of the participants	−0.15	0.18
Sex	0.47	< 0.001**
Ethnicity	−0.07	0.52
Current living location	0.24	0.029*
Marital status	0.006	0.96
Education	0.20	0.07
Employment	0.27	0.016*
Income	0.18	0.11
Injury level	0.19	0.09
Completeness of injury	−0.09	0.43
Chronic disease	−0.10	0.34
Complication related to SCI	−0.22	0.05
Caregiver	0.06	0.59

*p value < 0.05; **p value < 0.001

Pearson’s correlation matrix of resilience and psychosocial factors, i.e., social support, self-efficacy, spirituality, and depressive mood, are shown in Table 4. A significant positive correlation was found between resilience and social support (*r* = 0.42, *p* < 0.001) and self-efficacy (*r* = 0.53, *p* < 0.001). A significant negative correlation was identified between resilience and depressive mood which indicates that the participants with higher depressive mood had lower resilience (*r* = − 0.50, *p* < 0.001). However, a non-significant weak negative association was found between resilience and spirituality (*r* = − 0.12, *p* > 0.05).

**Table 4:** Correlation matrix between resilience and psychosocial factors

Variables	1	2	3	4	5
1. Resilience	1				
2. Social support	0.42**	1			
3. Self-efficacy	0.53**	0.37*	1		
4. Spirituality	−0.12	−0.01	−0.02	1	
5. Depressive mood	−0.50**	− 0.31*	− 0.49**	−0.04	1

*p < 0.01; **p < 0.001

Finally, multiple hierarchical regression analysis was performed when demographic variables, i.e., sex, employment, and current living location, were entered into the first block. Self-efficacy, depressive mood, and social support were entered into the subsequent blocks (Table 5). Since a non-significant association was found between spirituality and resilience, spirituality was excluded in the regression analysis. The full regression model was able to explain 46% of the variance in resilience (adjusted *R*^*2*^ = 0.46, F (6, 75) = 12.53, *p* < 0.001). In the first block, sex, employment, and current living location accounted for 23% of the variance in resilience (adjusted *R*^*2*^ = 0.23, *F* (3, 78) = 8.99, *p* < 0.001). The addition of self-efficacy into the second block greatly increased the variance explained in resilience to 40%, which was a change of 17% (adjusted *R*^*2*^ change = 0.17, *p* < 0.001). Depressive mood was entered into the third block which explained the 5% additional variance in resilience (adjusted *R*^*2*^ change = 0.05, *p* < 0.001). The addition of social support into the fourth block explained the additional 1% of variance; nevertheless, the increment was not significant (adjusted *R*^*2*^ change = 0.01, *p* > 0.05). Overall, sex, self-efficacy, and depressive mood were statistically significant factors of resilience. Among those, sex showed a higher beta value (β *=* 0.31, *p* = 0.001) indicating that sex was the strongest factor of resilience in this study. Furthermore, self-efficacy was found as a significantly strong psychosocial factor of resilience (β *=* 0.28, *p* = 0.007), followed by depressive mood (β *=* − 0.24, *p* = 0.016).

**Table 5:** Hierarchical regression analyses determining resilience

Independent Variables	Block 1		Block 2		Block 3		Block 4		95% CI
	*β*	*p*	*β*	*p*	*Β*	*P*	*β*	*P*	
Sex (female/male)	0.41	< 0.001	0.38	< 0.001	0.35	< 0.001	0.31	0.001*	(3.66, 13.89)
Employment (unemployed/employed)	0.10	0.30	0.001	0.99	0.02	0.87	0.02	0.87	(−5.23, 6.16)
Current living location (rural/urban)	0.14	0.16	0.03	0.70	0.007	0.94	0.04	0.65	(−3.94, 6.28)
Self-efficacy			0.45	< 0.001	0.33	0.001	0.28	0.007*	(0.07, 0.40)
Depressive mood					−0.27	0.008	−0.24	0.016*	(−1.14, − 0.12)
Social support							0.16	0.09	(−0.03, 0.45)
Adjusted *R*^*2*^	0.23		0.40		0.45		0.46		
Adjusted *R*^*2*^ change			0.17**		0.05**		0.01		

Model: *R*^*2*^ = 0.50; Adjusted *R*^*2*^ = 0 .46; *F* (6,75) = 12.53; *p* < 0.001

*p value < 0.05; **p < 0.001

## Discussion

This study examined the role of psychosocial and demographic factors in determining resilience among Nepalese with earthquake-related SCI. Among psychosocial variables, self-efficacy and depressive mood significantly influenced resilience among the participants. In addition, sex, current living location, and employment of participants were also significantly related to resilience. Social support was significantly correlated; nevertheless, it could not account for the significant variance in resilience in the regression analysis. Interestingly, there was no significant association between resilience and spirituality of the study participants.

As mentioned, the study results found that self-efficacy and depressive mood had a significant association with resilience in the participants. Evidence from previous studies also revealed self-efficacy as one of the significant determinants of resilience among people with SCI [6, 13, 16]. Self-efficacy is a vital factor which motivates and reinforces individuals to adopt health-promoting behaviors [33]. Individuals who are highly self-confident of their own capabilities can better manage a stressful situation or adversity without experiencing any negative psychological consequences. A feeling of high self-confidence to have control over things that one desires can result in resilience [34].

Consistent with previous studies, depressive mood was related to low resilience among the participants in this study [6, 13, 16]. A depressive mood among people with SCI can develop as a result of long-term negative consequences such as restriction in mobility, difficulty in adjustment or re-integration into the community, financial loss, loss of independence in daily activities, chronic pain, and a perceived bias due to the disability [35, 36]. Individuals with SCI who experience depressive mood tend to diminish self-care activities, avoid eating and performing exercises, have increased risk of developing medical complications, visit the hospital more frequently, and develop suicidal ideas and thoughts which can, in turn, contribute to low resilience [37].

Similar with a previous study conducted among people with SCI in Australia, social support had no significant predictive role in resilience [13]. However, social support was found as one of the significant determinants of resilience among disaster survivors [18, 38]. According to Bandura (1998), social support is not a self-forming entity which can directly buffer against stressors. However, it indirectly fosters a coping ability or resilience by enhancing perceived efficacy. An individual requires a strong sense of efficacy in order to seek or obtain social support [34]. In addition, internal factors related to confidence in regulating thoughts or emotions result in resilience regardless of social support [22].

In this study, there was no significant association between spirituality and resilience. This finding is contrary to previous studies conducted among people with SCI, which reported a significant positive relationship between these two variables [11, 20]. It could be attributed to several reasons. In comparison, the differences in research settings, durations after sustaining injury, and causes of SCI could result in disparate findings since spirituality is considered dynamic and varies according to circumstances [39]. According to the compensating reciprocal causation model, it is complex to understand the role of spirituality from a cross-sectional study since spirituality is dynamic in nature [40]. Since the Nepalese participants experienced the traumatic events that occurred in 2015, it is difficult to identify whether the events developed spirituality or spirituality was affected by the events. Consequently, a cross-sectional design could have yielded non-significant results in the present study. In addition, the narrow scale range of the ISS that was used to measure spirituality in this study possibly caused difficulty for the participants to accurately identify the subtle changes in the scores and possibly led to a lack of sensitivity to measure spirituality [11].

Among the demographic variables, sex significantly explained resilience in this study which was congruent with a study conducted among survivors of an earthquake in China [18]. However, previous studies conducted among people with SCI did not support this finding [6, 22]. The inconsistencies on the role of sex on resilience possibly resulted from the differences in the contexts and cultures of the studies. Cultural context influences a gender role and priorities, their sense of self-worth, and availability of social resources [41]. Nepal is a patriarchal society, where power or authority is held by males and they are considered to be the breadwinner in the family [42]. Nepalese males have higher employment and education opportunities and incomes compared to the females [24]. Due to the important roles of males in Nepalese society, they tend to receive support from their family and society. Consequently, a sense of worth or self-esteem is greater among the Nepalese males, including the disabled. Additionally, in the Nepalese context, females are treated as secondary to males [42]. Females with a disability are less likely to get married and have a family than males with a disability since those females are considered incapable of accomplishing the reproductive activities [43]. As a result, females tend to have a low sense of self-worth [41–43]. Thus, higher self-esteem, education, and employment opportunities among males could be the reason for higher resilience among the male participants as suggested in the literature [44, 45].

The results of this study also revealed that the current living location and employment were significantly associated with resilience. In Nepal, the majority of people live in rural areas where transportation services are inaccessible due to steep mountains and hills. Healthcare and rehabilitation services are available mostly in the urban areas. It takes several hours for people (including people with SCI) living in rural Nepal to access healthcare and rehabilitation services [24]. Inaccessibility to rehabilitation and treatment for complications related to SCI further deteriorate their physical health. Physical impairments could enhance the risk of developing depression and low self-efficacy among people with SCI [44] which subsequently contribute to low resilience among people living in rural areas [6].

This study supported the results of previous studies, which found that employed individuals tended to have significantly greater resilience than unemployed individuals [17, 45, 46]. People with SCI who are employed do not have to rely financially on others for survival which enhances their sense of self-worth and satisfaction with life which further enhances resilience [36, 45].

### Implications of the findings

The findings from this study offer several clinical implications for healthcare professionals, in particular, the rehabilitation nurses as well as mental health professionals. Identification of factors influencing resilience help rehabilitation nurses to plan appropriate strategies. For instance, self-efficacy strengthening interventions are beneficial to enhance resilience. Similarly, prevention or treatment of depressive mood is significant in promoting resilience of this group. Involvement of family members during the rehabilitation or nursing intervention need to be considered since social support is associated with resilience in this group. The study findings also suggest that nurses should identify or screen for low resilient groups such as females, the unemployed, rural people, and people with low self-efficacy, low social support, and unstable or depressed mood. Therefore, they can deliver interventions or care considering those vulnerable people. Community or home-based rehabilitation programs and nursing interventions would be effective since people from rural areas were found to be more vulnerable.

### Limitations and future research

Despite the significance of this study in understanding the factors of resilience following earthquake-related SCI, some limitations were identified and areas for possible future study could be suggested. First, the researcher read the questions and filled the questionnaires for illiterate participants which possibly developed some biases. The participants were likely to give desirable answers rather than answering their own feelings. Next, since this cross-sectional study was conducted at two years after the earthquake, it could not identify the dynamic nature of resilience and other variables. A longitudinal study is recommended to assess the long-term changes in resilience and factors influencing resilience. Although there is evidence that supports spirituality as a determinant of resilience, it did not make a contribution to resilience in the present study. Hence, further research related to spirituality using other measures need to be conducted. Furthermore, this study did not address the pre-injury mental health status or characteristics of the participants which could influence the post-injury mental health status or resilience of those individuals. Finally, testing the psychometric properties of the resilience instrument (CD-RISC) is important to examine the feasibility and sensitivity of the tool among Nepalese.

## Conclusion

Previous research findings were supported and this study reported more insight into resilience following earthquake-related SCI. The results indicated that self-efficacy, social support, and depressive mood were the significant psychosocial factors of resilience. Furthermore, demographic factors such as sex, employment, and living location were also associated with resilience. Therefore, interventions or rehabilitation should be focused on specific psychosocial factors as well as demographic factors. Future research regarding resilience interventions can aid rehabilitation nurses as well as mental health professionals to improve rehabilitation outcomes, enhance resilience, and promote successful reintegration into the community.

## Abbreviations

CD-RISC: Connor-Davidson Resilience Scale
CI: Confidence interval
ISS: Intrinsic Spirituality Scale
MSES: Moorong Self-efficacy Scale
MSPSS: Multidimensional Scale of Perceived Social Support
PHQ-9: Patient Health Questionnaire-9
SCI: Spinal cord injury
SIRC: Spinal Injury Rehabilitation Center
